# Free radical scavenging and antibacterial effects of pigskin peptide-functionalized NU-1000 composites

**DOI:** 10.1039/d6ra03326a

**Published:** 2026-07-17

**Authors:** Zhihui Jiang, Dandan Gong, Zimeng Liu, Xinning Ma, Jia Cao, Min Gao, Jing Yan

**Affiliations:** a Shandong University of Traditional Chinese Medicine Jinan 250355 P. R. China jingyan_2020@163.com

## Abstract

Pigskin peptides (PSPs) are bioactive collagen hydrolysates known for their antioxidant, antibacterial, and other biomedical potentials; however, their direct application is limited by poor environmental stability. Herein, we report the use of NU-1000, a robust zirconium-based metal–organic framework (MOF), as a delivery platform. A PSP-loaded NU-1000 composite, denoted as PSPs-1@NU-1000-2, was successfully constructed using PSPs with a molecular weight below 3000 Da, and this composite exhibited superior bioactivity. Structural and morphological characterization by FTIR, SEM, XRD, and BET confirmed the successful incorporation of PSPs into NU-1000. Notably, compared with free PSPs, the PSPs-1@NU-1000-2 composite demonstrated significantly enhanced DPPH radical scavenging activity and stronger antibacterial effects against *Escherichia coli* and *Staphylococcus aureus*. This MOF-based immobilization strategy not only preserves but also potentiates the bioactivities of PSPs, offering a promising peptide-nanomaterial composite for applications in the cosmeceutical, pharmaceutical, and food industries.

## Abstract 2

PSPs-1@NU-1000 was fabricated by immobilizing antioxidant pigskin peptide PSPs-1 within NU-1000. XRD confirmed successful incorporation while preserving crystallinity. The composite showed strong antibacterial activity against *E. coli* and *S. aureus*.

## Graphical abstract

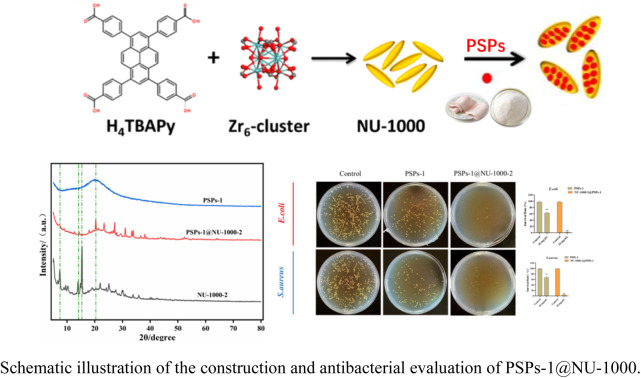

## 1 Introduction

Pigskin peptides (PSPs) are an important by-product of the porcine slaughtering industry, accounting for approximately 10% of the carcass weight, yet they remain far from fully exploited. This underutilization stems from the prevailing industrial model, which continues to focus on swine breeding and meat sales rather than on unlocking the potential value of such by-products. Studies have shown that porcine skin consists of 70% water, 28% protein, 0.6% ash, chondroitin sulfate, and other components, with collagen accounting for 87.7% of its total protein content.^1^ Biohistological analyses have revealed structural similarities and substantial molecular homology between porcine and human skin; moreover, collagen peptides derived from porcine skin exhibit exceptional biocompatibility and favorable absorption capacity.^2^

In addition, as typical bioactive peptides, Pigskin peptides possess diverse biological functions, including antioxidant,^3–6^ blood pressure-regulating,^7,8^ antibacterial,^9–11^ and mineral absorption-promoting activities.^12–14^ Further research on these bioactive substances is expected not only to enhance the added value of the pig industry chain but also to drive innovative applications of such raw materials in the food, pharmaceutical, and cosmetic sectors. However, peptide products are highly susceptible to oxidative degradation and loss of bioactivity upon direct exposure to the external environment;^15^ furthermore, free peptides may diffuse throughout the body and fail to accumulate at target sites, thereby reducing therapeutic efficacy and increasing the risk of side effects. In recent years, many researchers have therefore sought efficient drug delivery materials to improve the utilization of peptides in formulations.^16^

Metal–organic frameworks (MOFs), a class of porous hybrid materials fabricated *via* the self-assembly of metal nodes and organic ligands, have attracted significant research interest in the biomedical field due to their distinctive nanoscale structural properties.^17^ Among the vast MOFs, NU-1000 mesoporous zirconium-based MOF assembled from Zr_6_ nodes and tetratopic carboxylate ligands—stands out with several distinctive advantages that are rarely found in conventional microporous MOFs or other mesoporous analogues.^18,19^ First, its hierarchical three-dimensional porous network features large hexagonal mesochannels and smaller triangular microchannels, which not only provide sufficient spatial accommodation for bulky biomacromolecules but also enable size-selective encapsulation.^20^ Second, unlike many MOFs that suffer from poor stability in aqueous or physiological environments, NU-1000 exhibits exceptional hydrolytic and chemical robustness, ensuring structural integrity during biomolecule loading and release processes. Third, the abundant terminal hydroxyl groups on its Zr_6_ nodes serve as versatile reactive handles for postsynthetic functionalization, allowing facile surface engineering without compromising the underlying framework. Despite these favorable attributes, the application of NU-1000 as a carrier for natural antioxidants has not been previously reported. In this work, we construct a PSPs-1@NU-1000 composite by immobilizing the antioxidant pigskin peptide(PSP) within the NU-1000 scaffold. This design is conceptually innovative in three aspects. (i) The confinement effect of the NU-1000 mesopores not only achieves a high drug loading capacity but also effectively restricts the molecular motion of PSPs-1, thereby significantly enhancing its long-term antioxidant stability, which is a critical issue for free peptide that suffer from rapid activity decay. (ii) The intrinsic degradability of the Zr-based framework in physiological media enables a sustained and controlled release profile of the loaded PSPs-1, offering a programmable delivery platform. (iii) More importantly, beyond serving as a passive host, the Zr_6_ clusters themselves possess inherent bioactive properties—including the promotion of microcirculation and anti-aging effects—thus conferring the composite with synergistic antioxidant and broad-spectrum antibacterial functionalities.^21^

In this study, fresh pigskin was employed as the raw material for preparing pigskin peptides (PSPs) *via* a sequential process including pretreatment, degreasing, enzymatic hydrolysis, ultrafiltration, and freeze–drying. A highly bioactive fraction, PSP-1, with a molecular weight below 3000 Da was further isolated and screened. Concurrently, a series of NU-1000 materials were synthesized from ZrOCl_2_·8H_2_O and other precursors using different co-solvents, among which NU-1000-2 was selected as the optimal candidate due to its superior loading efficiency. The loading procedure of PSP-1 onto NU-1000-2 was optimized using single-factor tests combined with orthogonal array design, and the maximum loading efficiency of the resulting NU-1000-2@PSP-1 composite was determined to be 20.30%. The composite was subsequently characterized structurally, and its antioxidant and antibacterial properties were systematically evaluated.

## 2 Materials and methods

### 2.1 Experimental raw materials and reagents

Fresh porcine skin were purchased from Jiajiayue Supermarket in University Town, Changqing District, Jinan City, Shandong Province. All reagents were purchased from Sigma-Aldrich (St Louis, MO, USA). *Esclierichia coli* CCARM 1009 (*E.coli*), *Staphylococcus aureus* (*S. aureus*) were purchased from Guangdong Huan Kai Biotechnology Co., Ltd.

### 2.2 Preparation of porcine skin peptides

#### 2.2.1 Enzymatic hydrolysis of porcine skin peptides

Approximately 1000 g of fresh porcine skin was collected, and the adherent subcutaneous adipose tissue was carefully removed using a sterile scalpel. The dermal tissue was then minced, thoroughly rinsed with double-distilled water, and stored at 4 °C. After lyophilization for 24 h, the dried material was pulverized, and the resulting porcine skin powder was stored at −80 °C until further use.

For defatting, the lyophilized powder was suspended in eight volumes of 2% Na_2_CO_3_ solution, sonicated for 40 min, and incubated in a water bath at 40 °C for 4 h under magnetic stirring. The suspension was filtered through gauze, and the residue was rinsed repeatedly with water until neutral pH was achieved, yielding defatted porcine skin material.

Subsequently, the defatted material was suspended in eight volumes of ultrapure water, heated at 85 °C for 15 min, and then cooled to 40 °C. The pH was adjusted to 1.5–2.0 with 20% HCl, and pepsin was added at 2% (w/w, based on substrate mass) for hydrolysis at 40 °C for 2 h. Thereafter, the pH was adjusted to 7.5–8.0 using 20% NaOH, and trypsin was added at 2% (w/w) for further hydrolysis at 40 °C for 2 h.

Upon completion of enzymatic digestion, the hydrolysate was heated in boiling water for 10 min to inactivate the enzymes and then centrifuged at 5000×*g* for 10 min. The supernatant was collected and subjected to ultrafiltration using a membrane with a molecular weight cut-off (MWCO) of 3000 Da to obtain fractions with molecular weights of <3000 Da and >3000 Da. Each fraction was separately concentrated under reduced pressure and lyophilized for 24 h to obtain porcine skin peptide powders.

#### 2.2.2 Identification of peptide sequences by LC-MS/MS

An appropriate amount of enzymatic hydrolysate was reconstituted in 0.1% aqueous formic acid, vortex-mixed thoroughly, and centrifuged at 12 000 rpm for 10 min. The resulting supernatant was collected for subsequent LC-MS/MS analysis.

##### 2.2.2.1 Chromatographic separation

Peptides were separated on an Acclaim PepMap C18 analytical column (150 mm × 75 µm, 2 µm). Mobile phase A consisted of 0.1% formic acid in water, whereas mobile phase B consisted of 0.1% formic acid in acetonitrile. The flow rate was maintained at 300 nL min^−1^. Gradient elution was performed as follows: 0–5 min, 5% B; 5–45 min, 5–30% B; 45–50 min, 30–80% B; 50–55 min, 80% B; and 55–60 min, 80–5% B.

##### 2.2.2.2 Mass spectrometric analysis

Mass spectrometric detection was conducted using a nano-electrospray ionization source (nano-ESI). The spray voltage was set at 1.8 kV, and the ion transfer tube temperature was maintained at 250 °C. Full-scan MS spectra were acquired over an *m*/*z* range of 350–3000. MS/MS fragmentation was performed by higher-energy collisional dissociation (HCD) with a normalized collision energy of 27%. Dynamic exclusion was set to 30 s.

##### 2.2.2.3 Database search and peptide identification

The acquired tandem mass spectrometry data were searched against the UniProt database for peptide sequence identification. The database search parameters were configured as follows: trypsin was selected as the proteolytic enzyme, with a maximum of two missed cleavages permitted; peptide length was restricted to 3–25 amino acid residues; peptide confidence was set to >95%; and the false discovery rate (FDR) was controlled at <1%.

#### 2.2.3 Screened potential bioactive peptides based on their physicochemical properties

Potential bioactive peptides were screened based on their predicted bioactivity and physicochemical properties. The detailed procedure was as follows: peptide sequences identified by LC-MS/MS were subjected to *in silico* evaluation of bioactivity, toxicity, allergenicity, and solubility, and candidate peptides with potential biological activity were selected for further analysis.

##### 2.2.3.1 Screening of potential bioactive peptides based on peptide ranker

The bioactivity potential of the 135 peptides identified by LC-MS/MS was evaluated using the online tool PeptideRanker. PeptideRanker employs a machine-learning-based algorithm to predict the likelihood of peptide bioactivity, generating scores ranging from 0 to 1. Higher scores indicate a greater probability of biological activity. Peptides with PeptideRanker scores >0.5 were considered to have potential bioactivity and were retained for subsequent screening.

##### 2.2.3.2 Screening of non-toxic peptides based on ToxinPred

Peptides retained after bioactivity screening were further evaluated for potential toxicity using the online tool ToxinPred. ToxinPred predicts peptide toxicity based on a support vector machine algorithm and provides both prediction scores and classification results. Only peptides classified as “Non-Toxin” were selected for further analysis.

##### 2.2.3.3 Screening of non-allergenic peptides based on AlgPred2

The non-toxic peptides were subsequently subjected to allergenicity prediction using the online tool AlgPred2. AlgPred2 integrates multiple predictive approaches, including machine learning, MERCI motif-based prediction, BLAST similarity search, and hybrid methods, to assess the allergenic potential of peptide sequences. Peptides classified as “Non-Allergen” were retained for further evaluation.

##### 2.2.3.4 Analysis of peptide physicochemical properties based on PepCalc

The physicochemical properties of the non-toxic and non-allergenic peptides were analyzed using the online tool PepCalc. Parameters including molecular weight, isoelectric point, number of amino acid residues, net charge, and predicted solubility were calculated. Based on these results, peptides exhibiting favorable aqueous solubility were selected as candidate bioactive peptides.

#### 2.2.4 Infrared spectroscopy characterization of pigskin peptides

Pigskin peptides with distinct molecular weights were characterized *via* Fourier-transform infrared spectroscopy (FT-IR).

#### 2.2.5 Performance assay of pigskin peptides

##### 2.2.5.1 Antioxidant capacity of pigskin peptides with different molecular weights

###### 2.2.5.1.1 DPPH radical scavenging assay

5.0 mL of 15.0 mg mL^−1^ PSPs samples of different molecular weights were respectively set as *A*_1_ with 5.0 mL of 2,2-diphenyl-1-1-bitteryl hydrazine (DPPH) anhydrous ethanol solution, and the absorbance was measured at 517 nm. When anhydrous ethanol is used instead of DPPH solution, it is *A*_2_; when distilled water is used instead of the extract, it is *A*_3_; and when VC solution is used instead of the extract, it is *A*_0_. DPPH radical was calculated as follows:1
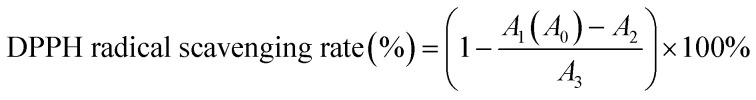


###### 2.2.5.1.2 Hydroxyl radical (·OH) scavenging assay

Add 8.0 mL of PBS buffer, 0.8 mL of 8.0 mmol L^−1^*o*-dinitrogen solution, 0.8 mL of 8.0 mmol L^−1^ ferrous sulfate solution, 1.6 mL of PSPs solutions of different molecular weights (15.0 mg mL^−1^), 3.2 mL of distilled water and 1.6 mL to the centrifuge tubes in sequence mL of 0.1% H_2_O_2_ solution, thoroughly mixed, set as *B*_1_, and the absorbance was measured at 536 nm. When distilled water is used instead of hydrogen peroxide solution, it is *B*_2_; when distilled water is used instead of the extract, it is *B*_3_; when VC solution is used instead of the extract, it is *B*_0_. Calculate the clearance rate of hydroxyl radicals based on formula (2). OH radical scavenging rate was calculated as follows:2
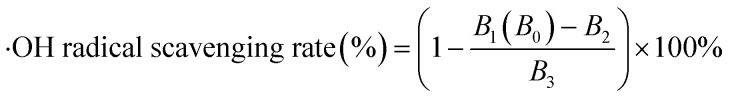


##### 2.2.5.2 Antibacterial activity of pigskin peptides with different molecular weights

###### 2.2.5.2.1 Preparation of culture medium

The medium used in this experiment was LB medium, with the formula as follows: tryptone (5.0 g), yeast extract (2.5 g), and sodium chloride (5 g) were dissolved in ultrapure water, and the volume was adjusted to 500 mL with ultrapure water. A 200 mL aliquot of the resulting solution was supplemented with 3 g agar powder to prepare solid medium. Then the medium was subjected to autoclaving at 121 °C for 1 h. Upon cooling to 60–70 °C, the agar-supplemented medium was dispensed into Petri dishes and stored for subsequent use.

###### 2.2.5.2.2 Reparation of the strain^23^

Aliquots of cryopreserved *Escherichia coli* CCARM 1009 (abbreviated as *E. coli*) and *Staphylococcus aureus* (abbreviated as *S. aureus*) were retrieved from a −80 °C ultra-low temperature freezer and inoculated into liquid medium-containing test tubes. Inoculants were resuscitated at 30 °C for 18 h in a shaking incubator, then spread-plated on solid medium and incubated at 30 °C for 24 h. Single colonies were isolated, resuspended in appropriate medium to prepare bacterial suspensions for subsequent experiments.

###### 2.2.5.2.3 Antibacterial activity assay

The photocatalytic antibacterial activities of the samples against *Escherichia coli* and *Staphylococcus aureus* were evaluated using the plate colony-counting method. Briefly, 200 µL of bacterial suspension was inoculated into a conical flask containing 30 mL of liquid culture medium and incubated in a constant-temperature shaker at 37 °C and 170 rpm for 12 h. The cultured bacterial suspension was then collected and diluted 100-fold.

For sample preparation, 0.01 g of each sample was dispersed in 1 mL of sterile water to obtain an aqueous dispersion with a concentration of 10 mg mL^−1^. Subsequently, 10 µL of the sample dispersion and 990 µL of the diluted bacterial suspension were added to the corresponding wells of a 24-well plate, yielding a final sample concentration of 100 µg mL^−1^. The experimental design included a blank control group containing only bacterial suspension and an experimental group containing both bacterial suspension and sample dispersion.

After treatment, the bacterial suspensions from the corresponding wells were serially diluted by 10^4^-fold with liquid culture medium. Then, 150 µL of the diluted bacterial suspension was uniformly spread onto the surface of solid agar medium. The plates were allowed to stand in a laminar flow hood for 20 min and then inverted and incubated at 37 °C for 36 h. After incubation, the plates were photographed, and the number of bacterial colonies was counted. The bacterial survival rate was calculated based on the colony-counting results. All experiments were performed in triplicate.

### 2.3 Synthesis of PSPs@NU-1000

#### 2.3.1 Synthesis of H_4_PyTBA

**Fig. 1:**
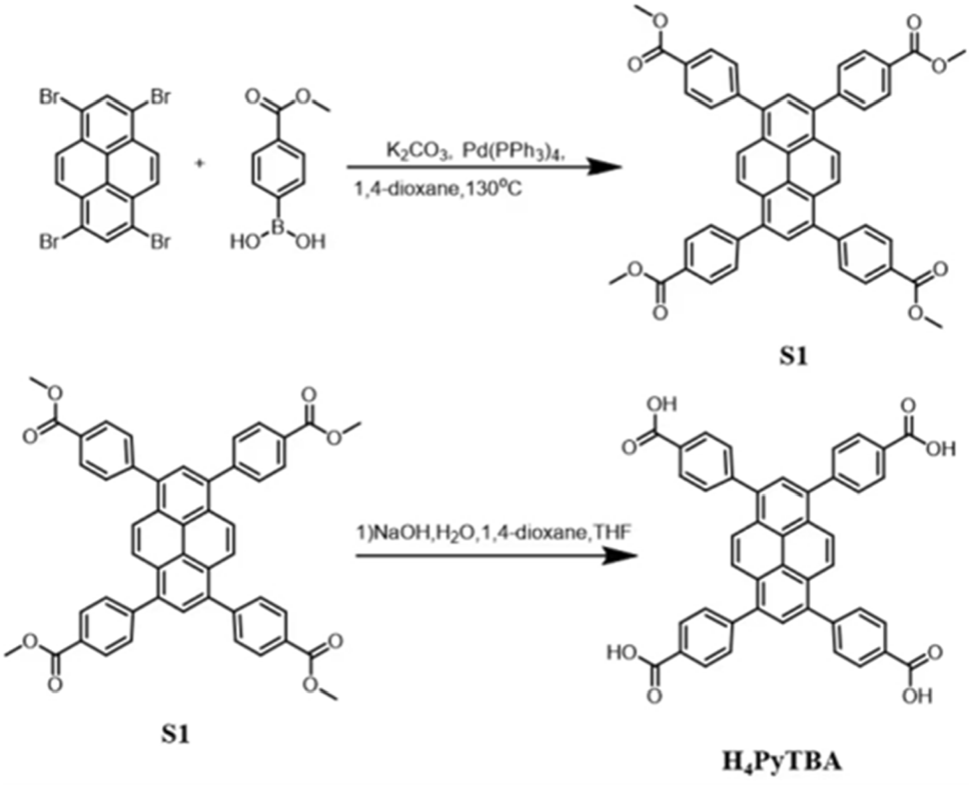
Synthesis for H_4_PyTBA.

1,3,6,8-Tetrabromopyrene (1.16 g, 2.24 mmol), 4-methylphenylboronic acid (2.4 g, 13.44 mmol), Pd (PPh_3_)_4_ (40 mg, 0.036 mmol), anhydrous potassium carbonate (0.6 g 4.34 mmol) and refined dioxane (100 mL), were heated at 130 °C in a heat-gathering constant-temperature magnetic stirrer and stirred for 78 hours. After cooling to room temperature, the reaction mixture was poured into a solution of ice water and concentrated hydrochloric acid (v/v = 3 : 1), stirred and filtered to obtain the cake. The filter cake was extracted with chloroform and water, and the combined extract was dried with anhydrous Na_2_SO_4_. The solvent was vacuum removed in a rotary evaporator to obtain a yellow solid. Add it to a single-mouthed flask, add 100 mL of suspension with a volume ratio of THF/dioxane/H_2_O (v/v = 5 : 2:2), add NaOH (1.04 g, 26.04 mmol) to the mixture, and reflux stir at 85 °C for 12 hours. After cooling, the solvent was removed under vacuum. Add 100 mL of H_2_O to the reactants and stirred at room temperature for 2 h. The pH of the system was adjusted to 2 with 6 mol L^−1^ concentrated hydrochloric acid, and stirring was continued for 30 min. Then yellow solid was collected by suction filtration and washed several times with water. High yellow solid H_4_PyTBA was obtained by vacuum drying after recrystallization of DMF (Fig. 1).^22^

#### 2.3.2 Synthesis of NU-1000

**Fig. 2:**
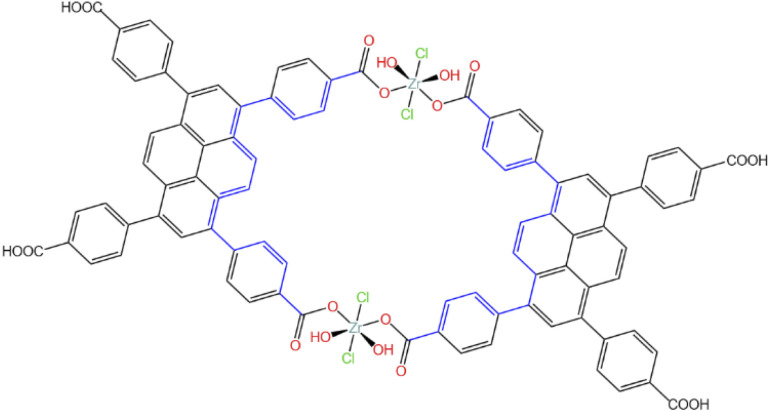
Synthesis of NU-1000.

Dissolve 0.2 mmol of ZrOCl_2_·8H_2_O and 22 mmol of 4-aminobenzoic acid (NH_2_-BA) in 8 mL of *N*,*N*-dimethylformamide (DMF), then the mixture reacted at 100 °C for 1 h in a heat-collecting constant-temperature magnetic stirrer. After cooling to room temperature, 0.06 mmol of H_4_PyTBA was added, followed by 5 µL of trifluoroacetic acid (TFA) and 1.0 mL of *N*,*N*-diethylformamide (DEF). The mixture was heated to 120 °C and continuously stirred in a heat-collecting constant-temperature magnetic stirrer for 1 hour. Then wash the product twice with anhydrous ethanol and dry it. Replace the DEF solvent with *N*,*N*-dimethylacetamide (DMA), octadecene (ODE), tetrahydrofuran (THF) and isopropanol (IPA) respectively, other synthetic procedures remained unchanged, yielding NU-1000-1 to NU-1000-5 (Fig. 2).^24^

#### 2.3.3 Synthesis of PSPs@NU-1000

20 mg of NU-1000 powder was dispersed in 10 mL of an aqueous PSPs solution (1.0 mg mL^−1^ in deionized water). The resulting suspension was incubated under shaking at 30 °C for 24 h in the dark to facilitate the adsorption/loading of PSPs onto NU-1000. Subsequently, the solid product was collected by centrifugation at 5000 rpm for 10 min, washed with ultrapure water to remove unbound PSPs, and the supernatant was collected for further analysis.^25^

#### 2.3.4 Determination of PSPs loading efficiency on NU-1000

Given the high abundance of hydroxyproline in porcine skin collagen, the loading efficiency of pigskin peptides was quantified using a hydroxyproline assay kit. Briefly, the samples were subjected to acid hydrolysis to release free hydroxyproline, which was subsequently oxidized by chloramine-T. The resulting oxidation products reacted with *p*-dimethylaminobenzaldehyde to produce a purplish-red chromophore. The hydroxyproline content was then determined by measuring the absorbance at 560 nm using a microplate reader. Based on the quantified hydroxyproline concentration, the loading efficiency of PSPs was calculated according to the following equation:^26^3
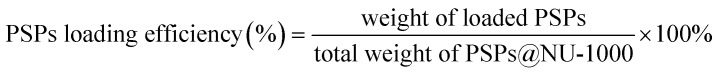


#### 2.3.5 Molecular dynamics simulation^27–33^

##### 2.3.5.1 System construction

The structure of the peptide was generated using AlphaFold, which yielded a high-confidence prediction (pLDDT = 87.83). The NU-1000 framework was constructed as a 2 × 2 × 2 supercell with dimensions of 7.85 × 7.85 × 3.31 nm^3^. For adsorption modeling, the peptide was initially placed 2.0 nm above the center of the largest pore of NU-1000. The system was then solvated in a periodic box of 7.85 × 7.85 × 8.31 nm^3^ using TIP3P water, corresponding to a solvent density of approximately 1000 kg m^−3^.

The peptide was described with the AMBER14SB force field, while the NU-1000 framework was parameterized using GAFF. To obtain reliable partial charges for the MOF, a representative NU-1000 fragment was optimized at the B3LYP/def2-TZVP level in ORCA 6.0, followed by RESP charge fitting.

##### 2.3.5.2 Molecular dynamics simulation

All MD simulations were performed with GROMACS 2026.2. Energy minimization was first carried out using the steepest descent method. The system was subsequently equilibrated through 200 ps *NVT* and 500 ps *NPT* simulations, during which positional restraints were imposed on the heavy atoms of both the peptide and NU-1000. The temperature and pressure were stabilized at 300 K and 1 atm, respectively.

A 200 ns production run was then conducted with a 2 fs time step. Long-range electrostatic interactions were treated using the PME method with a cutoff of 1.0 nm. The v-rescale thermostat and C-rescale barostat were used to regulate temperature and pressure, respectively. Periodic boundary conditions were.

##### 2.3.5.3 Analysis

Trajectory analyses were carried out using the built-in tools in GROMACS and the gmx_MMPBSA 1.6.5 package. The structural stability and conformational behavior of the peptide during adsorption were evaluated by calculating the root mean square deviation (RMSD) and root mean square fluctuation (RMSF) of the backbone atoms. Changes in peptide compactness were characterized using the radius of gyration (*R*_g_), whereas solvent exposure was quantified by the solvent-accessible surface area (SASA).

To probe the intermolecular interaction mechanism, nonbonded interaction energies between the peptide and NU-1000 were extracted from the production trajectories using the rerun protocol. The electrostatic (coulombic) and van der Waals (Lennard-Jones) contributions were analyzed separately to determine their respective roles in the adsorption process. In addition, MM/GBSA calculations were performed using gmx_MMPBSA to further decompose the binding energetics and quantify the contributions of different energy terms to peptide adsorption on the MOF framework. applied in all directions.

## 3 Results and discussion

### 3.1 Characterization and performance assay of porcine peptides

#### 3.1.1 Screened potential bioactive peptides based on their physicochemical properties

Potential bioactive peptides were screened based on their predicted bioactivity and physicochemical properties, five potential bioactive peptides were finally identified. Based on NU-1000 pore-size-dependent adsorption efficiency and considering DPPH radical scavenging rate, sequence 1 was identified as the most suitability for assembly onto NU-1000. To ensure a more comprehensive experimental design, a selected as PSP-2 for subsequent optimization experiments (Table 1).

**Table 1:** Potential bioactive peptides based on their physicochemical properties

No.	Sequence	Molecular weight (g mol^−1^)	pI	Analysis	DPPH radical scavenging rate (%)
1	TAGPSGPSGLPGER	1282.36	6.55	Near-neutral; relatively shortest	72.52
2	SGDRGETGPAGPAGPVGPVGAR	1962.09	6.67	Near-neutral; longest	72.11
3	GDRGEAGPAGPAGPAGPR	1589.67	6.93	Near-neutral; moderate molecular weight	63.62
4	DRGETGPAGPAGPVGPVGAR	1817.96	6.97	Closest to neutral, moderate molecular weight	70.09
5	HGDQGAPGPVGPAGPR	1469.56	7.57	Near-neutral, His-containing	45.02

#### 3.1.2 Infrared spectroscopy characterization of porcine peptide

**Fig. 3:**
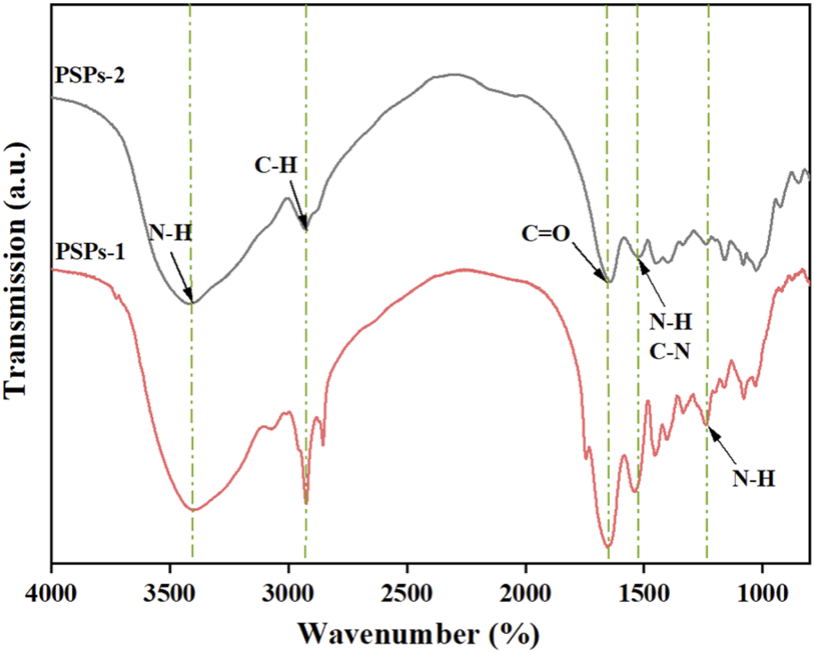
FT-IR spectra of PSPs-1 and PSPs-2.

FT-IR spectroscopy revealed that both PSPs-1 and PSPs-2 exhibited characteristic amide A band at 3419 cm^−1^ (attributed to the combination of N–H stretching vibration and hydrogen bonding), amide B band at 2927 cm^−1^ (C–H stretching vibration), strong amide I band at 1651 cm^−1^ (C=O stretching vibration, confirming carbonyl groups), amide II band at 1534 cm^−1^ (coupling of N–H bending and C–N stretching vibrations), and amide III band at 1245 cm^−1^ (deformation peak of N–H stretching vibration). These data were highly consistent with literature reports, confirming the enzymatic hydrolysis process can generate structurally intact collagen (Fig. 3).

#### 3.1.3 Antioxidant analysis of PSPs-1 and PSPs-2

**Fig. 4:**
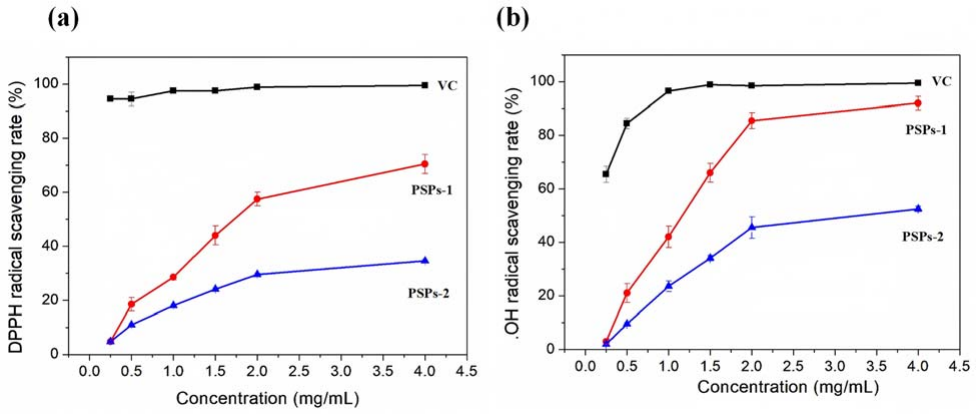
Antioxidant activity of *V*_c_ and PSPs-1, PSPs-2 against DPPH (a) and ·OH (b) radicals from 0.25 to 4 mg mL^−1^.

Fig. 4 respectively illustrate the PSPs-1, PSPs-2 against DPPH and ·OH radicals from 0.25 to 4 mg mL^−1^. As depicted in Fig. 4, both PSPs-1 and PSPs-2 exhibited concentration-dependent DPPH and ·OH radical scavenging capacities, far weaker than positive control Vc. PSPs-1 showed markedly stronger antioxidant activity than PSPs-2 under identical concentrations for both radicals. Scavenging rates rose rapidly at low concentrations and grew slowly with further concentration increase, showing saturated antioxidant tendency.

Therefore, PSPs-1 was selected for the subsequent loading experiments and antibacterial performance evaluations.

### 3.2 Characterization of NU-1000 and PSPs-1 loading efficiency on NU-1000

#### 3.2.1 Infrared spectral characterization of NU-1000

**Fig. 5:**
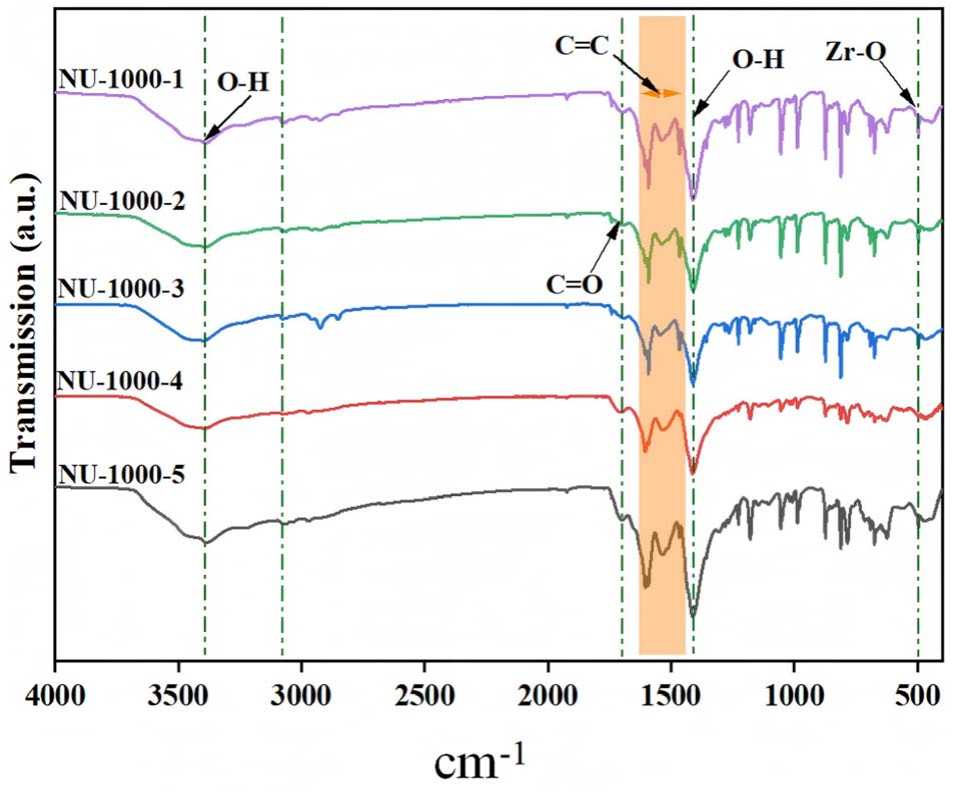
FT-IR spectra of NU-1000.

Infrared spectroscopy spectra shown in Fig. 5, there is a wide absorption peak in the 3477–3292 cm^−1^ region, which is the characteristic absorption peak of the carboxyl group. This spectral peak is generated by the stretching vibration of the O–H bond of the carboxyl group. The strong absorption peak at 1698 cm^−1^ is generated by the vibration of the C=O bond of the carboxyl group in the carboxylic acid. The strong absorption peak at 1415 cm^−1^ indicates the in-plane bending vibration of the O–H bond. These three sets of peaks suggest that the NU-1000 prepared in different solvents has a carboxyl structure. Besides, there is an absorption peak at 3070 cm^−1^, which is the stretching vibration of the C–H bond on the benzene ring. Four-finger peaks of varying intensities appear at 1602 cm^−1^, 1589 cm^−1^, 1536 cm^−1^, and 1463 cm^−1^. Thus, there is a mononuclear aromatic C=C skeleton, and confirmed the existence of benzene rings. The peak at 498 cm^−1^ is generated by the Zr–O bond, which indicates that the structure of the NU-1000 prepared under different solvents was correct.

#### 3.2.2 PSPs-1 loading efficiency on NU-1000

The determination of PSPs-1 loading efficiency was performed following Section 2.3.4 and the results of NU-1000 synthesized using different solvents shown in Table 2. NU-1000-2 showed the highest loading efficiency for PSPs-1, so NU-1000-2 was selected for the process optimization experiment.

**Table 2:** Loading efficiency of NU-1000

Samples	Loading efficiency (%)
NU-1000-1	8.8
NU-1000-2	34.8
NU-1000-3	0.7
NU-1000-4	1.8
NU-1000-5	5.1

### 3.3 Optimization of PSPs loading onto NU-1000 matrix

#### 3.3.1 Single-factor experiments on PSPs loading onto NU-1000

This part investigated the optimal process conditions of NU-1000 loading with PSPs. Taking the loading efficiency of PSPs as the evaluation index, the effects of three single factors (oscillation time, oscillation temperature and material-peptide ratio) on PSPs loading efficiency were investigated, and an orthogonal experiment was designed to optimize the loading process.

##### 3.3.1.1 Effect of oscillation duration on the loading efficiency of PSPs onto NU-1000

10 mg of NU-1000-2 was added to 10 mL of PSPs-1 aqueous solution (1.0 mg mL^−1^ in deionized water) at a material-peptide ratio of 1 : 3, and 25 °C oscillation temperature. The effect of reaction time was investigated at time points of 1 h, 6 h, 12 h, 24 h, and 30 h.

##### 3.3.1.2 Effect of oscillation temperature on the loading efficiency of PSPs onto NU-1000

10 mg of NU-1000-2 was added to 10 mL of PSPs-1 aqueous solution (1.0 mg mL^−1^ in deionized water) at a material-peptide ratio of 1 : 1 and the oscillation time of 12 h. The effect of reaction temperatures on PSP loading efficiency was investigated at different reaction temperatures: 15 °C, 25 °C, 35 °C, 45 °C, 55 °C.

##### 3.3.1.3 Effect of material-peptide ratio on the loading efficiency of PSPs onto NU-1000

PSPs-1 and NU-1000-2 were mixed at a material-peptide ratio of 2 : 1, 1 : 1, 1 : 2, 1 : 3 and 1 : 4, respectively, with oscillation time fixed at 12 h and temperature at 35 °C.

#### 3.3.2 Results of single-factor experiments

**Fig. 6:**
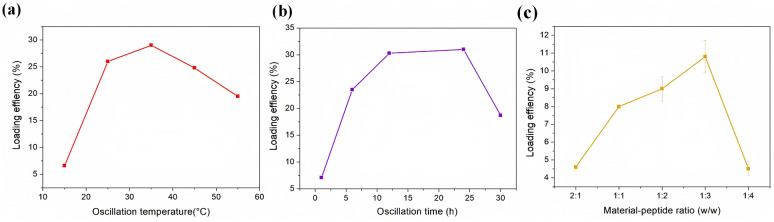
(a) The effect of oscillation time on the load rate of NU-1000 load PSPs; (b) the effect of oscillation temperature on load rate of NU-1000 load PSPs; (c) the effect of peptide ratio on load rate of NU-1000 load PSPs.

As shown in Fig. 6a, loading efficiency increased with prolonged oscillation time, peaking at 24 h; further extension induced a decline. Nevertheless, the loading efficiency at 12 h was only 1% lower than that at 24 h. Therefore, an oscillation time range of 6–18 h was selected for subsequent experiments based on comprehensive considerations. As shown in Fig. 6b, the loading efficiency increased with rising temperature, peaking at 35 °C; further temperature elevation induced a decline. Thus, an oscillation temperature range of 25–45 °C was selected for subsequent experiments. As shown in Fig. 5c, the loading efficiency increased with increasing peptide-to-material ratio, peaking at a ratio of 1 : 3; a further increase in the ratio induced a decline. Thus, a material-peptide ratio range of 1:2–1:4 was selected for subsequent experiments.

#### 3.3.3 Orthogonal array experiments

Based on the above single-factor experimental results for PSPs loading onto NU-1000, oscillation temperature (°C), oscillation time (h), and peptide-to-material mass ratio (w/w) were selected as independent variables, and experiments were designed according to L_9_(3^4^) orthogonal array to determine the optimal process for PSPs loading onto NU-1000. The orthogonal experimental design is shown in Table 3, the corresponding results presented in Table 4.

**Table 3:** Factor and level of orthogonal test for PSPs loading onto NU-1000

Level of factor	Oscillation temperature (°C)	Oscillation time (h)	Material-peptide ratio (w/w)
1	25	6	1 : 2
2	35	12	1 : 3
3	5	18	1 : 4

**Table 4:** Intuitive analysis table for orthogonal test of NU-1000-based PSPs loading

Laboratory no.	Oscillation temperature (°C)	Oscillation time (h)	Material-peptide ratio (w/w)	Load rate (%)
1	1	1	1	5.48
2	1	2	2	13.66
3	1	3	3	9.24
4	2	1	2	9.86
5	2	2	3	20.30
6	2	3	1	16.00
7	3	1	3	2.35
8	3	2	1	8.63
9	3	3	2	13.22
*K*_1_	28.38	17.69	30.11	
*K*_2_	46.16	42.59	36.74	
*K*_3_	24.20	38.46	31.89	
*k*_1_	9.46	5.90	10.04	
*k*_2_	15.39	14.20	12.25	
*k*_3_	12.83	12.82	10.63	
*R*	7.32	8.30	2.21	

The orthogonal experimental results showed that oscillation time (*R* = 8.30) > oscillation temperature (*R* = 7.32) > material-peptide ratio (*R* = 2.21). Because a larger range value (*R*) indicates a more significant effect of the factor on loading efficiency, oscillation time exerted the most prominent influence on PSP loading onto NU-1000, followed by oscillation temperature, while material-peptide ratio had the least effect. The optimal level combination is: oscillation temperature: *k*_2_ (15.39), the largest selected 35 °C; oscillation time: *k*_2_ is the maximum (14.20), select 12 hours. Material-peptide ratio: *k*_2_ is the largest (12.25). The optimal condition is: temperature: 35 °C, oscillations time: 12 h, material-peptide ratio: 1 : 3.

### 3.4 Characterization of PSPs-1@NU-1000-2

#### 3.4.1 Infrared spectra of PSPs-1@NU-1000-2

**Fig. 7:**
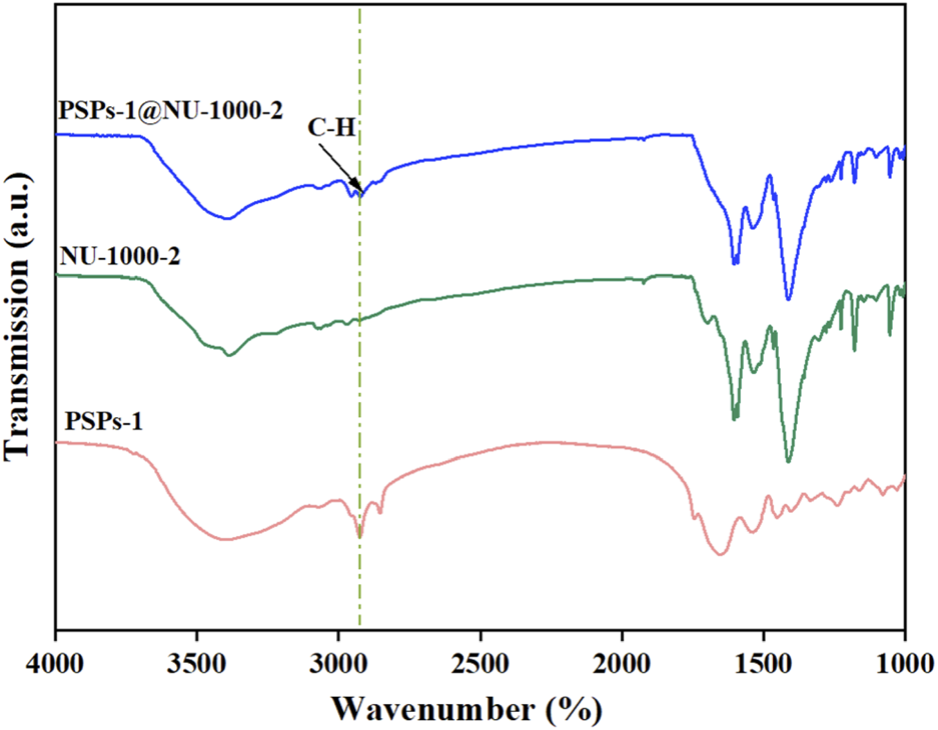
Infrared spectra of PSPs-1, NU-1000-2, and PSPs-1@NU-1000-2.

The infrared spectra of the PSPs-1@NU-1000-2 was shown in Fig. 7. The absorption peak at 2927 cm^−1^ is generated by the stretching vibration of the C–H bond and belongs to the amide B band, which is a characteristic absorption peak of PSPs. This absorption peak does not exist in NU-1000 but appears in PSPs-1@NU-1000-2, indicating that PSPs has been loaded onto NU-1000.

#### 3.4.2 SEM and TEM characterization of PSPs-1@NU-1000-2

**Fig. 8:**
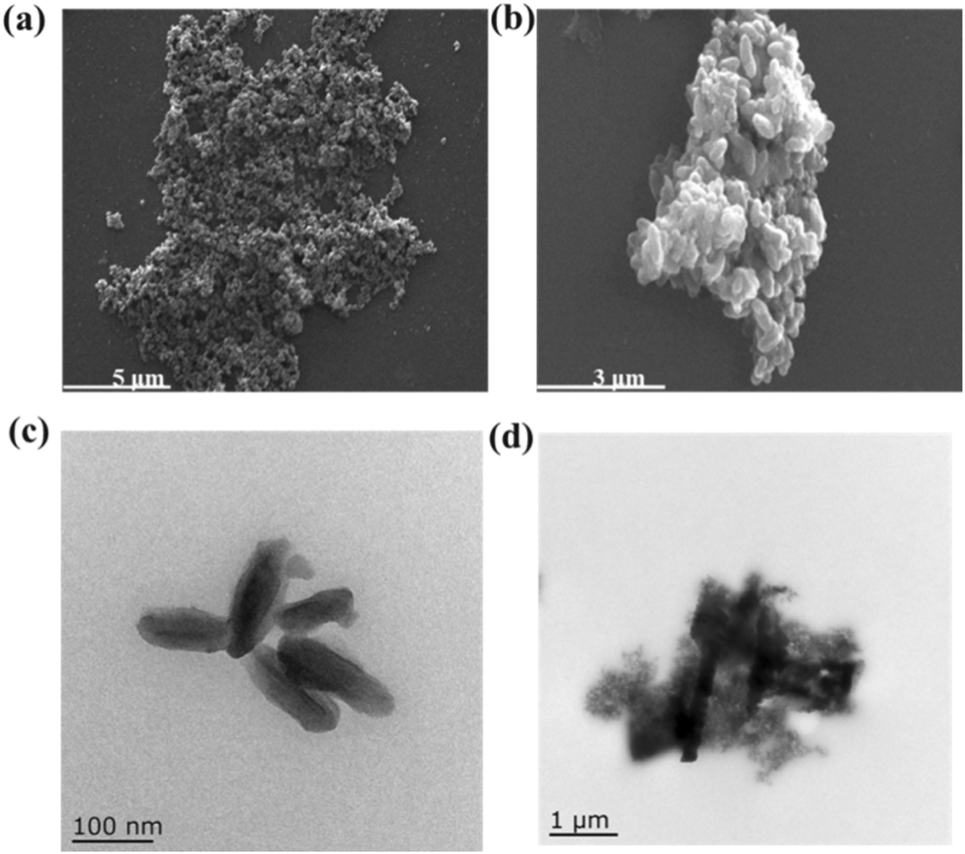
SEM images of NU-1000-2 (a), PSPs-1@NU-1000-2 (b); TEM image of NU-1000-2 (c), PSPs-1@NU-1000-2 (d).

The morphology and microstructure of PSPs-1@NU-1000-2 were further investigated by scanning electron microscopy (SEM) and transmission electron microscopy (TEM). As shown in Fig. 8a and b, pristine NU-1000-2 exhibits a well-defined hexagonal rod-like morphology with relatively smooth surfaces, consistent with the characteristic morphology of NU-1000. In contrast, PSPs-1@NU-1000-2 maintains the overall rod-like architecture but displays noticeably roughened surfaces accompanied by nanoscale deposits or coating layers, suggesting the successful incorporation or surface immobilization of PSPs-1 on the NU-1000-2 framework.

TEM observations provide additional evidence for the structural features of the composite. Pristine NU-1000-2 presents highly ordered mesoporous channels with clear lattice/channel contrast, indicative of its well-preserved crystalline porous structure (Fig. 8c and d). After PSPs-1 loading, the composite exhibits attenuated or partially blurred channel contrast in the peripheral regions, while the internal ordered framework remains largely intact. This phenomenon indicates that PSPs-1 is preferentially anchored on the external surface and within the outer pore regions of NU-1000-2 rather than causing collapse or significant disruption of the framework.

Collectively, the SEM and TEM results demonstrate the successful formation of PSPs-1@NU-1000-2, in which PSPs-1 is closely associated with the NU-1000-2 surface and outer mesoporous channels, giving rise to an intimate core-shell-like interfacial structure while preserving the intrinsic crystalline architecture of the NU-1000-2 scaffold.

#### 3.4.3 BET spectra of PSPs-1@NU-1000-2

**Fig. 9:**
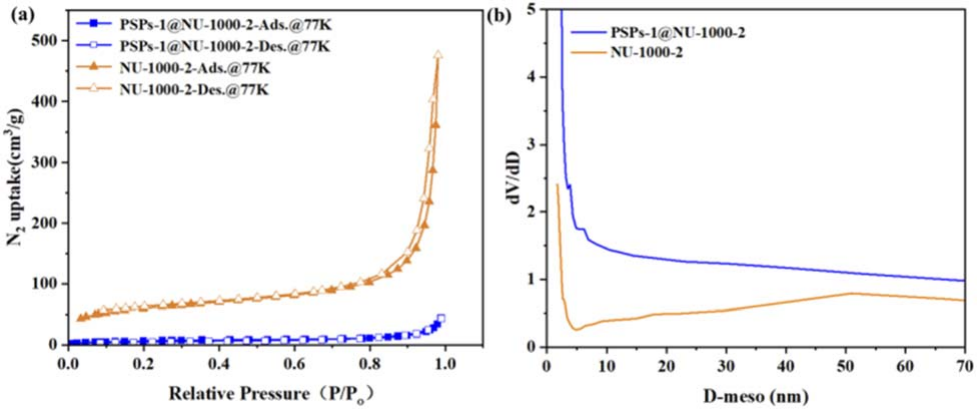
(a) N_2_ adsorption–desorption isotherms at 77 K and (b) pore size distribution profiles of bare NU-1000-2 and PSPs-1@NU-1000-2.

Fig. 9a shows the N2 adsorption–desorption isotherms of NU-1000-2 and PSPs-1@NU-1000-2 measured at 77 K, and Fig. 9b presents the corresponding pore size distribution curves. Pristine NU-1000-2 exhibits a high BET specific surface area of 2150 m^2^ g^−1^. After loading PSPs-1, the saturated N_2_ adsorption capacity of PSPs-1@NU-1000-2 is significantly decreased, and the BET specific surface area is reduced to approximately 200 m^2^ g^−1^. In addition, the mesopore size distribution shifts toward smaller pore diameters. These results indicate that PSPs-1 molecules are successfully confined within the mesoporous channels of NU-1000-2, thereby occupying part of the pore volume and leading to a marked reduction in both specific surface area and pore size.

#### 3.4.4 XRD patterns of PSPs-1@NU-1000-2

**Fig. 10:**
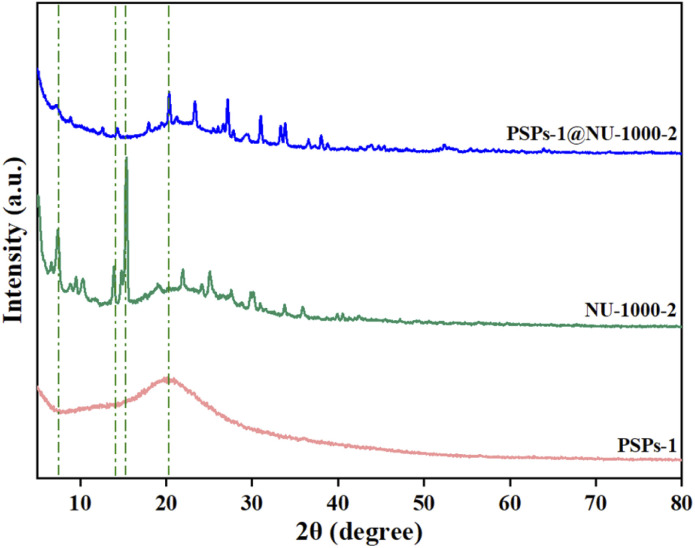
XRD spectra of PSPs-1, NU-1000-2, and PSPs-1@NU-1000-2.

The XRD pattern of PSPs-1@NU-1000-2 was shown in the Fig. 10. The peaks at 7.38°, 13.98° and 15.36° are characteristic diffraction peaks of MOFs materials, indicating that NU-1000-2 has a good crystal structure. The XRD pattern of PSPs-1 shows a broad “steamed bun peak”, which indicates that PSPs-1 has a typical amorphous structure. The XRD pattern of PSPs-1@NU-1000-2 shows a characteristic peak at 13.98°, while the main peak disappears at 15.36°, indicating a change in the crystal structure of NU-1000-2. Additionally, in PSPs-1@NU-1000-2, there is a new characteristic peak appears at 20.4°, which is the same as the XRD characteristic peak of PSPs-1. This result indicates that PSPs-1 has been successfully loaded on NU-1000-2. However, the disappearance of the main XRD peak and crystal structure alteration of NU-1000-2 could be ascribed to the macromolecular nature of PSPs-1. With a molecular size exceeding the pore diameter of NU-1000-2, PSPs-1 may stretch and fracture the pore walls during loading, disrupting the framework's long-range order and thereby eliminating the main diffraction peak.

#### 3.4.5 Molecular dynamics simulation of the PSPs-1@NU-1000

To further clarify the molecular basis of PSPs-1 loading onto NU-1000, molecular dynamics simulations were performed to examine the adsorption behavior, structural evolution, and interaction energetics of the peptide–MOF system. As shown in the simulation snapshots, PSPs-1 gradually approached the NU-1000 framework and was eventually accommodated within its large pore region, indicating a spontaneous adsorption process. The RMSD of the overall system reached a stable plateau after approximately 3 ns, suggesting rapid equilibration and stable maintenance of the adsorbed state. Meanwhile, RMSF analysis revealed distinct changes in peptide flexibility after adsorption: the C-terminal residues became less mobile, whereas the N-terminal electrostatic mechanism, providing molecular-level support for the successful construction of the PSPs-1@NU-1000 composite region exhibited increased flexibility, indicating that the interaction with NU-1000 altered the conformational dynamics of PSPs-1. Consistent with this observation, both the radius of gyration (*R*_g_) and solvent-accessible surface area (SASA) increased after adsorption, with SASA rising from approximately 13 nm^2^ to 16–17 nm^2^ during the initial stage of the simulation. These results indicate that PSPs-1 underwent adsorption-induced conformational extension on the NU-1000 surface, accompanied by increased solvent exposure. Energy analysis further demonstrated that the adsorption process was primarily driven by electrostatic interactions. The coulombic interaction energy was consistently much more favorable than the Lennard–Jones contribution throughout the simulation, and MM/GBSA calculations yielded a total binding free energy of −71.93 ± 22.27 kcal mol^−1^, confirming that peptide adsorption onto NU-1000 is thermodynamically favorable. Among the decomposed energy terms, the electrostatic contribution (Δ*E*_ELE = −135.04 ± 35.15 kcal mol^−1^) was the dominant driving force, whereas the van der Waals term (Δ*E*_VDW = −17.44 ± 4.30 kcal mol^−1^) and nonpolar solvation term (Δ*E*_SURF = −2.20 ± 0.74 kcal mol^−1^) contributed less significantly; although the polar solvation term (Δ*E*_GB = 82.74 ± 39.92 kcal mol^−1^) opposed binding, it was outweighed by the strong electrostatic attraction. Overall, these results indicate that PSPs-1 can be stably adsorbed within NU-1000 through a predominantly.

### 3.5 Performance of PSPs-1@NU-1000-2

#### 3.5.1 Antioxidant analysis of PSPs-1 and PSPs-1@NU-1000-2

The results of antioxidant activity were shown in Fig. 12. Fig. 12a and b display the DPPH and ·OH radical scavenging rates of free PSPs-1 and PSPs-1@NU-1000-2 composite over incubation times ranging from 0 to 48 h. The radical scavenging activity of pure PSPs-1 gradually declined as the incubation time prolonged, while PSPs-1@NU-1000-2 maintained distinctly higher scavenging efficiency against both radicals at all time points. These results demonstrate that loading PSPs-1 onto NU-1000 effectively improves the stability and long-term antioxidant capacity of PSPs-1.

**Fig. 11:**
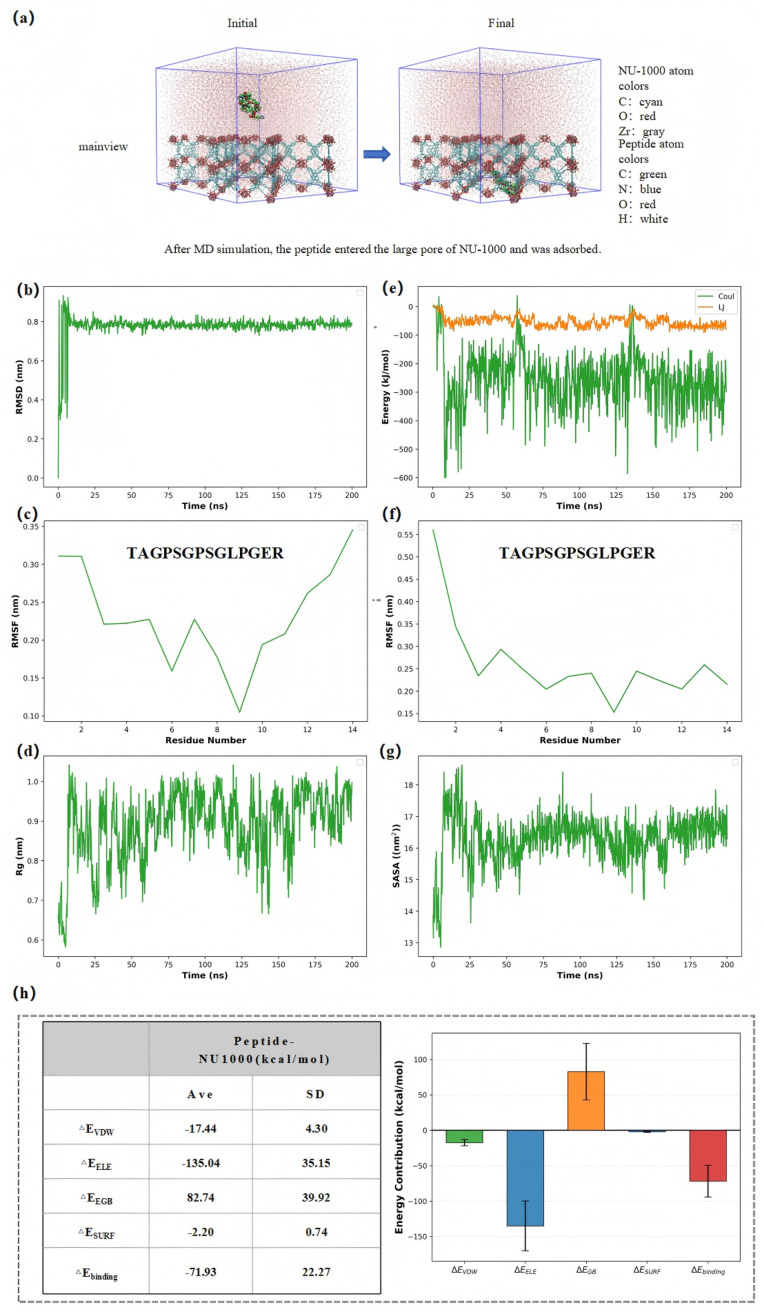
MD simulation of the PSPs-1@NU-1000 system. (a) Initial and final adsorption configurations. (b) RMSD of the system. (c and f) RMSF of PSPs-1 before and after adsorption. (d) *R*_g_ of PSPs-1. (e) Interaction energies between PSPs-1 and NU-1000. (g) SASA of PSPs-1. (h) MM/GBSA energy decomposition.

**Fig. 12:**
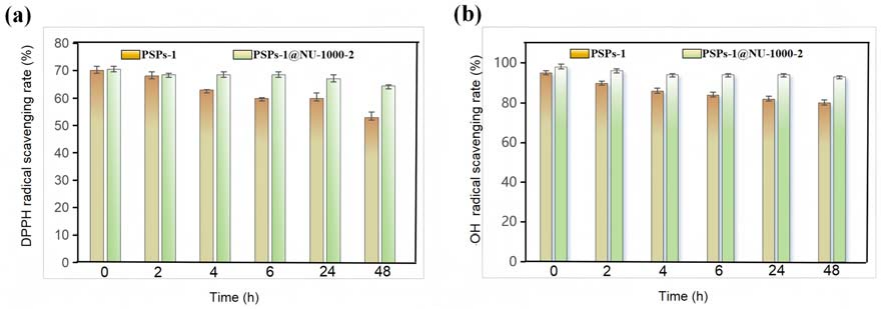
Antioxidant activity of PSPs-1 and PSPs-1@NU-1000-2 against DPPH (a) and ·OH (b) radicals from 0 to 48 h.

#### 3.5.2 Antibacterial activity

**Fig. 13:**
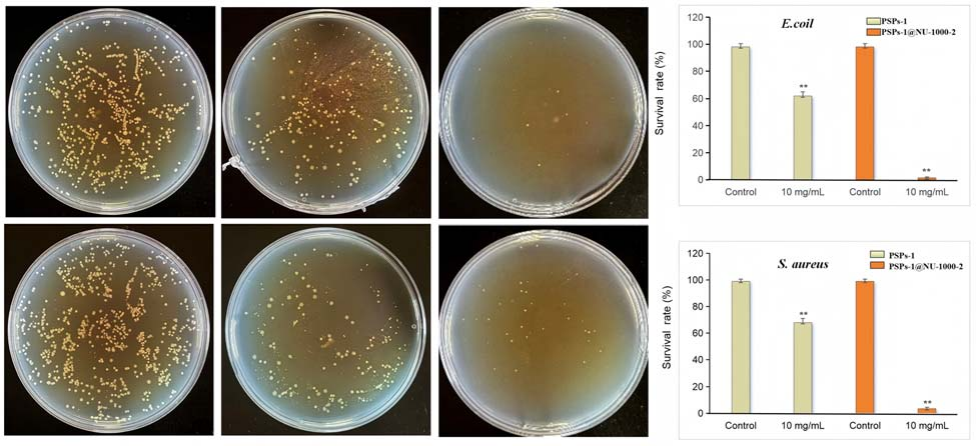
Representative colony images and bacterial survival rates of *E. coli* and *S. aureus* treated with blank control, PSPs-1 and PSPs-1@NU-1000-2.

Fig. 13 illustrates the antibacterial performance of PSPs-1 and PSPs-1@NU-1000-2 against *E. coli* and *S. aureus via* the plate colony counting assay, with quantitative bacterial survival rates presented in the accompanying bar graphs. The blank control group formed abundant, dense bacterial colonies for both strains, confirming normal bacterial growth without sample treatment. PSPs-1 displayed moderate antibacterial activity, leading to a marked drop in colony counts; the bacterial survival rates reached 60% for *E. coli* and 69% for *S. aureus*. Remarkably, the PSPs-1@NU-1000-2 composite displayed drastically enhanced antibacterial efficacy: almost no visible bacterial colonies were observed on the agar plates, with bacterial survival inhibition rates reaching 99% for *E. coli* and 98% for *S. aureus*. These findings indicate that immobilizing PSPs-1 onto NU-1000 significantly boosts its antibacterial activity against both Gram-negative and Gram-positive bacteria, achieving a near-complete bactericidal effect.

##### 3.5.2.1 Screening of antibacterial activity of PSPs-1 and PSPs-1@NU-1000-2

The antibacterial activities of PSPs-1 and PSPs-1@NU-1000-2 were further evaluated using the standard inhibition zone assay. A 9.0 cm diameter agar plate was used, and inhibition zone diameters corresponding to 50%, 70%, 80%, and 90% of the plate diameter were adopted as reference criteria for evaluating antibacterial performance.

As shown in Table 5, PSPs-1 exhibited an inhibition zone diameter of 5.600 ± 0.289 cm against *Escherichia coli*, corresponding to an inhibition rate of 62%. For *Staphylococcus aureus*, the inhibition zone diameter of PSPs-1 was 6.200 ± 0.267 cm, corresponding to an inhibition rate of 69%. In comparison, PSPs-1@NU-1000-2 showed markedly enhanced antibacterial activity. The inhibition zone diameter against *E. coli* reached 8.900 ± 0.328 cm, corresponding to an inhibition rate of 99%, while that against *S. aureus* was 8.400 ± 0.127 cm, corresponding to an inhibition rate of 93%.

**Table 5:** Inhibition zone diameter of PSPs-1 and PSPs-1@NU-1000-2 against *E. coli* and *S. aureus*

Samples	Diameter of the antibacterial zone (cm)	
	*E. coli*	*S. aureus*
PSPs-1	5.600 ± 0.289	6.200 ± 0.267
PSPs-1@NU-1000-2	8.900 ± 0.328	8.3400 ± 0.127

All experiments were performed in triplicate, and the data are expressed as mean ± standard deviation(SD). The inhibition zone assay demonstrated that the inhibition rates of PSPs-1@NU-1000-2 against both *E. coli* and *S. aureus* exceeded 93%, which were substantially higher than those of pristine PSPs-1 (62–69%). These results are consistent with the MIC/MBC results and further confirm that immobilization within NU-1000 effectively enhances the bactericidal activity of PSPs-1.

##### 3.5.2.2 MIC and MBC determination of PSPs-1 and PSPs-1@NU-1000-2

The minimum inhibitory concentration (MIC) and minimum bactericidal concentration (MBC) of PSPs-1 and PSPs-1@NU-1000-2 were determined using the LB broth dilution method. A series of sample concentrations, including 160.00, 80.00, 40.00, 20.00, 10.00, 5.00, 2.50, 1.25, and 0.625 mg mL^−1^, were prepared for antibacterial evaluation.

Briefly, different concentrations of PSPs-1 and PSPs-1@NU-1000-2 were separately added into 48-well plates, followed by the addition of bacterial suspensions of *E. coli* or *S. aureus*. The mixtures were thoroughly mixed and incubated at 37 °C. The MIC was defined as the lowest concentration at which visible bacterial growth was inhibited after incubation. To determine the MBC, 100 µL aliquots from wells showing no obvious turbidity were spread evenly onto LB agar plates and incubated at 37 °C for 24–48 h. The MBC was defined as the lowest concentration at which no bacterial colonies were observed on the agar plates. Each experiment was independently repeated three times.

As shown in Table 6, both PSPs-1 and PSPs-1@NU-1000-2 exhibited stronger antibacterial activity against *S. aureus* than against *E. coli*. Moreover, the MBC/MIC ratios were all ≤4, indicating that these samples exerted bactericidal rather than merely bacteriostatic effects. After immobilization of PSPs-1 onto NU-1000, the MIC values against both bacterial strains were markedly reduced. Specifically, the MIC value against *S. aureus* decreased from 20 to 15 mg mL^−1^, while that against *E. coli* decreased from 40 to 20 mg mL^−1^. These results are consistent with the antibacterial-rate assay, in which the antibacterial efficiency of the composite increased from approximately 60% to 99% at a concentration of 10 mg mL^−1^. Collectively, these findings demonstrate that NU-1000 loading effectively improves the antibacterial and bactericidal efficacy of PSPs-1.

**Table 6:** MIC and MBC values of PSPs-1 and PSPs-1@NU-1000-2 against *E. coli* and *S. aureus*

Samples	*E. coli*		*S. aureus*	
	MIC (mg mL^−1^)	MBC (mg mL^−1^)	MIC (mg mL^−1^)	MBC (mg mL^−1^)
PSPs-1	40	100	20	60
PSPs-1@NU-1000-2	20	80	15	50

#### 3.5.3 Cytotoxicity assays

**Fig. 14:**
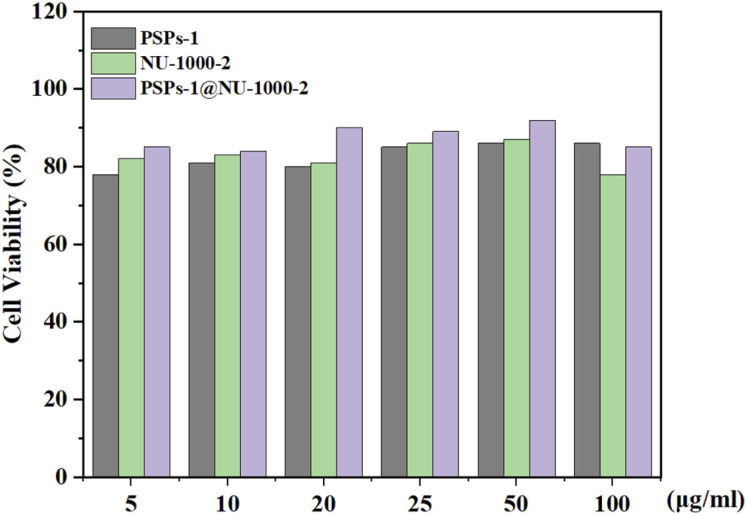
Cytotoxicity of PSPs-1, NU-1000-2 and PSPs-1@NU-1000-2.

Cytotoxicity assays were performed on PSPs-1, NU-1000-2 and PSPs-1@NU-1000-2, the results shown in Fig. 11. At concentrations ranging from 5 to 100 µg mL^−1^ (5, 10, 20, 25, 50, 100 µg mL^−1^), all three samples induced high cell viability, mostly above 80%. These results demonstrate that within the tested concentration range, PSPs-1, NU-1000-2 and PSPs-1@NU-1000-2 exhibit low cytotoxicity, negligible inhibitory effects on cell viability, and favorable biocompatibility (Fig. 14).

## 4 Conclusions

This study systematically investigated the process for loading PSPs onto NU-1000. With loading efficiency as the evaluation index, *N*,*N*-dimethylacetamide (DMA) was confirmed as the optimal solvent for NU-1000 synthesis. With antibacterial and antioxidant activities as screening criteria, PSPs with molecular weight <3000 Da were selected for their superior bioactivity. With loading efficiency of PSPs as the evaluation index, the effects of three single factors (oscillation time, oscillation temperature and material-peptide ratio) on PSPs loading efficiency were investigated, and an orthogonal experiment was designed to optimize the loading process. Besides, the antioxidant and antibacterial activities of the resultant composite, thereby providing additional evidence for its potential applications in antioxidant cosmeceuticals and antibacterial pharmaceuticals. The main conclusions are as follows:

(1) PSPs were extracted *via* enzymatic hydrolysis, and two molecular weights of PSPs (PSPs-1 and PSPs-2) were isolated by ultrafiltration. The results of FT-IR characterization proving that PSPs extracted by this enzymatic hydrolysis method had a complete structure. Then, antioxidant experiments and antibacterial experiments proving PSPs-1 yielded superior performance.

(2) MOF materials with different pore sizes were synthesized under different co-solvents. FT-IR characterization verified their structures, and NU-1000-2 exhibited the optimal performance which DMA was employed as the co-solvent.

(3) Single-factor experiments and orthogonal experiments were conducted to examine the effects of material-peptide ratio, oscillation temperature, and oscillation time on PSPs loading efficiency. The optimal conditions were material-peptide ratio 1 : 3, oscillation temperature 35 °C, and oscillation time 12 h. FTIR, SEM, XRD, and BET analysis confirmed the structure of PSPs-1@NU-1000-2. Then antioxidant and antibacterial assay of PSPs-1@NU-1000-2 were performed: DPPH radical scavenging rate is 53.42%, the ·OH clearance rate is 46.74%, the antibacterial rate of *E. coli* is 100%, the antibacterial rate of *S. aureus* is 98.59%. Antibacterial ability against *E. coli* and *S. aureus* of PSPs-1@NU-1000-2 were both superior to those of PSPs before loading onto NU-1000.

## Author contributions

All authors contributed to the conception and design of the study. Material preparation and data collection were performed by Zhihui Jiang and Dandan Gong. Data analysis was conducted by Zhihui Jiang, Xinning Ma, Jia Cao, and Min Gao. Revision experiments and additional data collection were carried out by Zimeng Liu. The first draft of the manuscript was written by Zhihui Jiang, and critical review and revision of the manuscript were provided by Jing Yan. Financial support was provided by J. Yan. All authors read and approved the final version of the manuscript.

## Conflicts of interest

There are no conflicts to declare.

## Data Availability

All data generated or analyzed during this study are included in this published article. For any additional raw data that support the findings of this study, they are available from the corresponding author upon reasonable request, without undue reservation.
